# Knowledge Mapping of Stem Cell Therapy for Premature Ovarian Insufficiency: A Bibliometric Analysis (2000-2023)

**DOI:** 10.2174/011574888X329310241206105808

**Published:** 2024-12-27

**Authors:** Yuting Cao, Jinyuan Huang, Xiaoyin Fan, Yinmei Dai

**Affiliations:** 1 Department of Gynecology, Beijing Obstetrics and Gynecology Hospital, Capital Medical University/Beijing Maternal and Child Health Care Hospital, Beijing, China;; 2 State Key Laboratory of Female Fertility Promotion, Center for Reproductive Medicine, Department of Obstetrics and Gynecology, Peking University Third Hospital / National Clinical Research Center for Obstetrics and Gynecology (Peking University Third Hospital) / Key Laboratory of Assisted Reproduction (Peking University), Ministry of Education / Beijing Key Laboratory of Reproductive Endocrinology and Assisted Reproductive Technology / National Clinical Key Specialty Construction Program, P. R. China (2023), Beijing, 100191, China

**Keywords:** Stem cells, therapy, premature ovarian insufficiency, VOSviewer, CiteSpace, bibliometrix

## Abstract

**Background:**

Premature Ovarian Failure (POI), a prevalent gynecological, endocrine disease, significantly impairs the reproductive health of women of childbearing age and presents a formidable challenge to clinicians. Until now, there has been a lack of effective treatments to fundamentally improve ovarian function in patients with POI. Stem cell therapy has emerged as a promising treatment in the field of POI, with notable research progress achieved to date.

**Objectives:**

This review sought to analyze the current status and hotspots of research on stem cell therapy for POI, forecasting future directions through bibliometrics.

**Methods:**

Research related to stem cell therapy for POI from 2000 to 2023 was searched in the Web of Science Core Collection (WOSCC) database by setting subject-term, and the literature was analyzed econometrically using VOSviewer, CiteSpace, and the R package “bibliometrix.”

**Results:**

According to our search and screening strategy, 203 pieces of literature related to stem cell therapy for POI were obtained and analyzed. There is a marked annual increase in publications, with a particularly rapid ascent in recent years. China has become the most prolific country in this field, with 136 publications. Shanghai Jiao Tong University ranked first among many universities and institutions in terms of the number of publications and citations. Stem Cell Research & Therapy was the most popular and influential journal in the field of stem cell therapy for POI. Lai Dongmei has published the most papers, while Liu Te boasts the highest frequency of co-citations. Investigation into the mechanisms of exosomes derived from stem cells and their associated signaling pathways is anticipated to be a crucial research topic in stem cell therapy for POI.

**Conclusion:**

This review offers the first comprehensive and systematic analysis of the field of stem cell therapy for POI, with a visual representation of the findings. By summarizing the current status and projecting forthcoming trends, this study aims to offer guidance and a reference for scholars in the field.

## 1 INTRODUCTION

Premature Ovarian Insufficiency (POI) is a challenging clinical syndrome defined as a reduction in ovarian function before the age of 40 years [1]. POI was first described in 1942, and its terminology has evolved since then, including terms such as premature menopause, Premature Ovarian Failure (POF), and primary ovarian insufficiency [2]. It is biochemically characterized by menstrual disturbance (amenorrhea or oligomenorrhea), hypergonadotropic conditions, and premature reduction in estrogen, significantly leading to infertility, vaginal dryness, and sleep disturbances [1-3]. The hypoestrogenic condition in patients with POI is also known to increase the risk of cardiovascular disorders, parkinsonism, depression, osteoporosis, hypertension, weight gain, midlife diabetes, cognitive decline, and dementia, contributing to a reduced quality of life [4, 5]. According to the European Society for Human Reproduction and Embryology (ESHRE) guideline diagnostic criteria, POI is diagnosed based on the presence of menstrual disturbance (oligo/amenorrhea for at least 4 months) and biochemical confirmation (an elevated FSH level >25 IU/l on two occasions >4 weeks apart) [2]. The prevalence of POI varies by region, ethnicity, lifestyle, and environment, affecting approximately 1% to 3.7% of women under 40 years old and 0.1% of women under 30 years old [3, 6, 7]. Multiple factors, including genetic abnormalities, autoimmunity, radiotherapy, chemotherapy, and surgery, can contribute to declining ovarian function, though the cause remains unknown in most cases [8].

Historically, the first-line treatment for POI has been hormone replacement therapy, which can relieve menopausal symptoms and improve quality of life but does not fundamentally restore ovarian function, including secretion, ovulation, and fertility [9, 10]. Clinically, there remains a lack of effective therapeutic options for POI. To preserve ovarian function, new therapeutic strategies, such as *in vitro* activation, mitochondrial activation technique, stem cell and exosome therapy, biomaterials strategies, and platelet-rich plasma intra-ovarian infusion, are being developed and becoming available for POI patients [11]. For those lacking residual follicles, stem cell therapy may represent a promising and clinically meaningful treatment option to induce oocyte generation in the emerging field of regenerative medicine [12].

Stem cell therapy, poised to transform 21st-century healthcare, has the ability to self-renew, proliferate indefinitely, and differentiate into multiple cell types, regenerating tissue based on environmental cues and signals. As such, it has been proposed for treating diseases, including POI [13-15]. In recent years, the mechanisms of stem cell therapy for POI have been explored, with the goal of advancing translational applications and clinical trials to cure POI. Stem cells exert their therapeutic effects by restoring endocrine ovarian function and fertility through homing, differentiation, paracrine stimulation, stem cell-mediated exosomes, and mitochondrial transfer, with paracrine stimulation improving damaged ovaries *via* anti-apoptosis, anti-fibrosis, angiogenesis, anti-inflammation, and immune regulation [8, 11]. With enormous literature on the pathophysiologic mechanism and treatment of stem cell therapy for POI, some scholars have shown significant interest in conducting related clinical trials, both domestically and internationally [16-21]. Considering the great breakthroughs in this field, numerous traditional systematic reviews have summarized the research progress on stem cell therapy for POI [5, 8-11]. However, traditional systematic reviews lack quantitative and comprehensive visual analysis, and the number of included studies has been limited, making it difficult to summarize advancements, analyze emerging trends, and improve systematization on a larger scale [22, 23]. Therefore, it is imperative to grasp the current status and trends of stem cell therapy for POI through bibliometrics.

Bibliometric analysis, an extensively recognized method of literature analysis, uses mathematical models and statistical methods to evaluate research characteristics both quantitatively and qualitatively, visualize the status and trends of scholarly literature within a specialized field, and predict forefront hotspots in global scientific research [24-26]. This analysis provides detailed information about authors, keywords, journals, productive countries and institutions, references, and funding agencies. It also helps identify collaborative relationships among authors, countries, and institutions and evaluate the academic contributions of these entities in the relevant research field [25, 27-29]. Several recent studies have carried out bibliometric analyses of stem cell therapy across multiple fields, including pulmonary fibrosis [30], stroke [31], spinal cord injury [32], liver diseases [33], glaucoma [34], cardiovascular disease [35] and so on. However, to the best of our knowledge, no bibliometric analysis of stem cell therapy for POI has been performed. Based on the fact that POI severely impacts the reproductive health of young women and stem cell therapy shows considerable therapeutic potential, we conducted a bibliometric analysis to quantify and visualize research on stem cell therapy for POI. Our goal was to clarify the current research status and provide valuable insights for further research by analyzing literature over the past 24 years.

## 2 MATERIALS AND METHODS

### 2.1 Literature Sources and Search Strategy

In terms of literature retrieval, Web of Science (WOS) [https://www.webofscience.com/wos/alldb/basic-search] is the most world-recognized and suitable database for bibliometric research. The documents in this study were retrieved from the WOS Core Collection (WOSCC) database based on subject-term searching on August 28, 2023. The detailed retrieval formula was utilized as follows: TS=(“Premature Ovarian Insufficiency” OR “Primary Ovarian Insufficiency” OR “Premature Ovarian Failure” OR “premature menopause”) AND TS=(“Stem cell” OR “Progenitor Cell” OR “mesenchymal stem cell”), with article language limited to English. The timespan for retrieval was set from January 1, 2000, to August 28, 2023, while the document types were limited to “articles” and “reviews.”

### 2.2 Inclusion and Exclusion Criteria

**Fig. (1):**
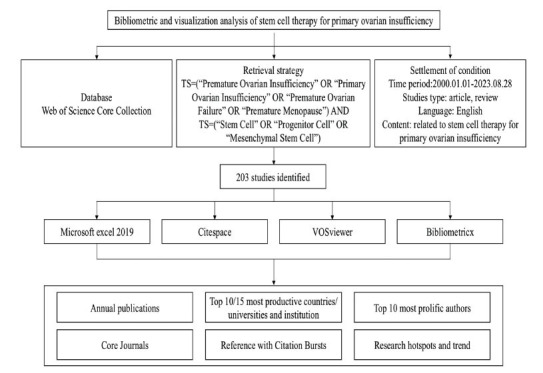
Flow chart of publications screening and selection process.

The inclusion and exclusion criteria of studies were firstly based on the filters of the WOSCC database and then manually screened. As described above, inclusion criteria were: 1] articles involving stem cells and POI, 2] articles published from a period spanning January 1, 2000, to August 28, 2023, 3] peer-reviewed published original articles or reviews, and 4] English publications included. Exclusion criteria were: 1] the duplicate publications, 2] meeting abstract, editorial material, early access, patent, letters, book chapters, correction, case report, retracted publication, reprint, and other types, 3] literature that did not address stem cell therapy for POI, or only addressed it rather than focusing on it by examining the title, abstract, and text in detail. The whole screening process was completed and agreed upon independently and strictly by Yuting Cao and Jinyuan Huang according to the inclusion and exclusion criteria. The screening process of literature was subjective, which may cause bias in selection and publication. The screening flow chart showed the search strategy, inclusion, and exclusion criteria of literature about stem cell therapy for POI from the WOSCC database and the process of the bibliometric analysis (Fig. **1**).

### 2.3 Data Collection and Cleaning

According to the aforementioned search strategy and inclusion criteria, a total of 498 potential articles and reviews were screened, with 203 available literature selected for subsequent analysis based on exclusion criteria. Each document contained the title, authors, abstract, keywords, country, organization, publication year, journal, references, citations, document type, and DOI. Notably, no ethical approval was required for this bibliometric analysis, as all the data were obtained from public databases and analyzed retrospectively without human participants [26]. All filtered data were initially downloaded as plain text for subsequent analysis and exported on the same day (August 28, 2023) to account for changes in metrics over time [23]. Before conducting the bibliometric analysis, synonymous keywords were merged. In addition, some data required cleaning and summarization. The data from Taiwan, the Macau Special Administrative Region, and the Hong Kong Special Administrative Region were grouped under China [26]. Journal category quartile rankings (Q1-Q4) and the Impact Factor (IF) were extracted from the Journal Citation Reports (JCR) report 2022 [https://jcr.clarivate.com/jcr/home].

### 2.4 Data Analysis

In this study, we used the JAVA program VOSviewer (version 1.6.19, Leiden University, Leiden, Netherlands), CiteSpace (version 6.2.R4, Drexel University, Philadelphia, USA), and the open-source tool Bibliometrix based on R programming language (https://www.bibliometrix.org/) for bibliometric analysis and network visualization, leveraging their complementary strengths. VOSviewer, primarily used to build collaboration, co-citation, and cooccurrence networks, was employed to analyze countries, universities and institutions, journals and co-citation journals, authors and co-citation authors, co-occurrence keywords, and co-citation references in the present study. In the knowledge maps produced by VOSviewer, items such as countries, universities, institutions, journals, authors, keywords, and references were represented as nodes. The size and color of these nodes reflected the proportion and classification of these items, respectively [36, 37]. The thickness of the connecting lines between different nodes represented the strength of collaboration, co-occurrence, or co-citations among them. Bibliographic co-citation and keyword co-occurrence networks were used to construct a knowledge map of stem cell therapy for POI. Reference co-citation cluster analysis and keyword co-occurrence analysis, respectively, illustrated the main topics in this field and identified key themes in the literature [23, 38]. Additionally, Microsoft Office Excel 2019 software was used to conduct the annual distribution trends of the literature. Compared to VOSviewer, CiteSpace offers advantages in visualizing dual-map overlays of journals to trace citing trajectories and performing the burst-citation analysis [39, 40]. Furthermore, Bibliometrix was used to perform visual analysis, focusing on two aspects: [1] mapping the global distribution and collaboration networks in stem cell therapy for POI research, and [2] understanding the development of the field, assessing the research status, and predicting the future hot spots based on keyword analysis in different periods.

## 3 RESULTS

### 3.1 Trend of Annual Publications in Stem Cell Therapy for POI

**Fig. (2):**
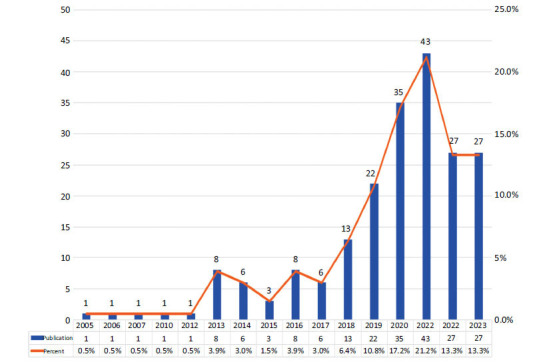
The distribution publications trend from January 1, 2000, to August 28, 2023.

According to the aforementioned retrieval strategy, there were a total of 203 studies of stem cell therapy for POI from January 1, 2000, to August 28, 2023 (Fig. **1**). Among these, 148 (72.91%) were indexed as articles, and 55 (27.09%) were indexed as reviews. The distribution of publications by year is shown in Fig. (**2**). The whole period of research on stem cell therapy for POI from 2000 to 2023 can be divided into three stages: Period I (2000-2012), Period II (2013-2017), and Phase III (2018-2023). During the early part of Period I (2000-2004), there were no publications, but in 2005, the world's first study on stem cell therapy for POI was published. However, not more thanone article was published per year from 2005 to 2012, indicating that research in this area was in its infancy. The number of publications in phase II remained in the single-digit stage and exhibited a stable trend with the average annual publication number of about 6.2, which was in the initial stage of this field. In phase III, the total number of publications began to increase significantly, with an average annual number of about 27.8, which was significantly higher than the other two stages. From 2018 to 2021 (within phase III), the number of publications related to stem cell therapy for POI demonstrated an upward trend year by year, reaching a peak in 2021 (n=43). Although fewer publications were recorded in 2022 compared to 2021 and 2020, the number of publications in the first eight months of 2023 equaled that of 2022, indicating that publication rates in 2023 may still improve and attract the attention of scholars (Fig. **2**).

### 3.2 Country Analysis

According to the search results, a total of 27 countries around the world contributed to the reported publications in the field of stem cell therapy for POI. As shown in Table **1**, the top 10 countries were distributed across Asia, North America, Europe, Africa, and Oceania, with a predominance of countries from Asia (n=5) and Europe (n=2). Among the various countries, the largest number of publications was from China (n=136), followed by the United States (n=25) and Iran (n=22), while the remaining published no more than six articles each. In terms of total citations, China had the most citations among nations (3995 citations), followed by the United States (618 citations), Iran (359 citations), Japan (160 citations), Egypt (148 citations), and South Korea (143 citations). As the tenth most prolific country, Japan had the highest average number of citations (40.00 times), followed by China (29.38 times), the United States (24.72 times), Egypt (24.67 times), and South Korea (23.83 times). Consequently, our analysis revealed that China is the most prominent country and far exceeded that of other countries, with the highest publications and citations, as well as ranking among the top two average citations.

**Fig. (3):**
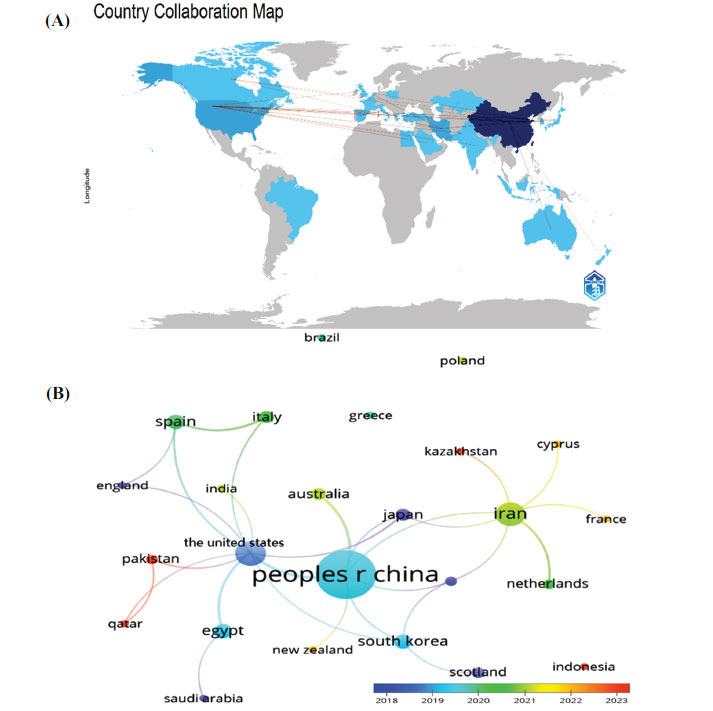
(**A**) The geographical distribution and (**B**) visualization of countries in stem cell therapy for POI.

Subsequently, based on a minimum of two publications and the relationships among countries, we visualized countries and constructed a collaborative network to illustrate the number of publications and cooperative relationships (Figs. **3A**, **B**). The size of the nodes represented the influence of the countries, while the thickness and distance of the connected lines between nodes represented the cooperative relationships between countries. As indicated in Figs. (**3A**, **B**), there was considerable active cooperation between different countries. For instance, China has developed extensive cooperation with other countries in the field of stem cell therapy for POI, particularly with the United States and Australia. In addition, Spain and Italy demonstrated active collaboration, while the United States also showed close cooperation with Egypt.

### 3.3 University and Institution Analysis

A total of 321 universities and institutions globally were involved in the research of stem cell therapy for POI. The top 15 institutions, each with five or more related publications, were distributed across four countries: Eleven in China, two institutions in the United States, one in Iran, and one in the Czech Republic (Table **2**). Ranking by the number of publications, the top 15 institutions collectively published 122 articles, accounting for 60.10% of the total, surpassing the combined output of other institutions. Among them, Shanghai Jiao Tong University contributed the maximum number of publications (18 publications), followed by Nanjing Medical University (16 publications), Binzhou Medical University (9 publications), and Chongqing Medical University (9 publications). The top 15 institutions in terms of total citation and average citation are also shown in Table **2**. Shanghai Jiao Tong University (837 citations) significantly outranked other institutions in this field, followed by Nanjing Medical University (573 citations), Chinese Academy of Sciences (342 citations), and Shanghai University of Traditional Chinese Medicine (289 citations). In addition, Shanghai Jiao Tong University achieved the highest average number of citations (46.50 times), followed by the Chinese Academy of Sciences (42.75 times), Shanghai University of Traditional Chinese Medicine (41.29 times), and Tongji University (41.00 times). As the most published institution, Shanghai Jiao Tong University ranked first in both the total number of citations and the average number of citations, far exceeding other organizations.

**Fig. (4):**
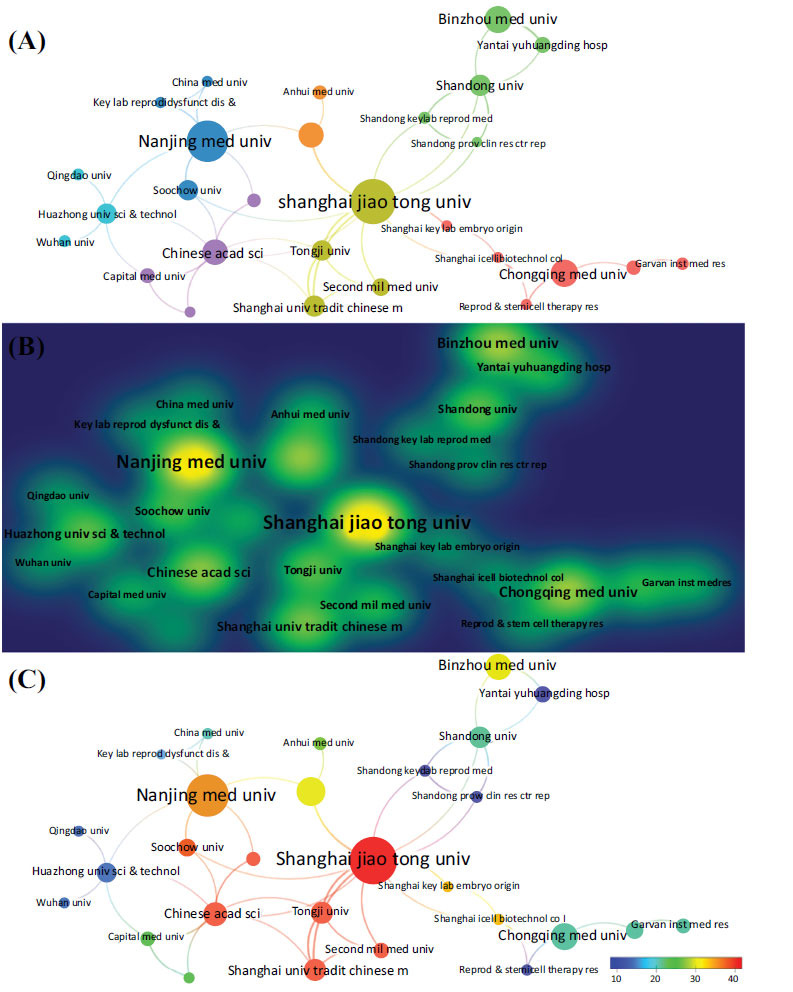
Visualization of universities and institutions. (**A**) Network diagram, (**B**) Density diagram, and (**C**) Reference intensity diagram.

Subsequently, we constructed a collaboration network of 66 institutions, based on a minimum publications threshold of two, excluding the 38 disconnected items, to visualize the number and relationships of publications among universities or institutions (Fig. **4**). The results showed the formation of four primary clusters, with the biggest node representing the institution that published the most in its cluster, including Shanghai Jiao Tong University, Nanjing Medical University, Binzhou Medical University, and Chongqing Medical University (Figs. **4A**, **B**). Furthermore, active cooperation was observed among Shanghai Jiao Tong University, Chinese Academy of Sciences, Shanghai University of Traditional Chinese Medicine, Tongji University, and Second Military Medical University (Fig. **4C**).

### 3.4 Journal and Co-citation Journal Analysis

This study found that studies on stem cell therapy for POI were published in 93 journals. The basic information on the top 12 journals with four or more publications is summarized in Table **3**, including category quartile rankings, IF, total citation, and average citation per journal. Around 48.77% (99/203) of the publications were published in the top 12 most influential journals. Among the top 12 journals, Stem Cell Research & Therapy (n=43) had the most publications, followed by Reproductive Sciences (n=9) and Stem Cell Reviews and Reports (n=7). Articles published in these three journals accounted for 29.06% of the total number of publications in this study. As category quartile rankings and IF are essential parameters for evaluating the quality and value of the journal, 2022 IF -about the top 12 journals ranged from 2.70 to 7.50, with Stem Cell Research & Therapy having the highest impact factor (IF=7.50), followed by Frontiers in Cell and Developmental Biology (IF=5.50) and Frontiers in Endocrinology (IF=5.20), while the rest had IF of no more than 5. In addition, it was found that the top 12 journals were mainly categorized within Q1 and Q2. Moreover, our analysis showed that the most highly cited journal was Stem Cell Research & Therapy (1624 citations), followed by Scientific Reports (243 citations) and Biomed Research International (197 citations). Furthermore, the journal with the highest average citations was Scientific Reports (60.75 times), followed by Biomed Research International **(**49.25 times) and Stem Cell Research & Therapy (37.77 times). Stem Cell Research & Therapy had the highest number of publications, the highest IF, and the highest total citations among the 12 journals, while Scientific Reports had the highest average citations, exceeding 60.

**Fig. (5):**
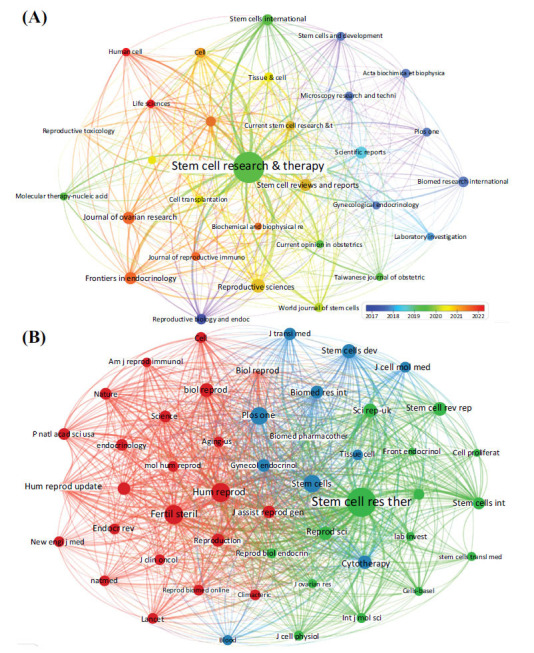
Visualization of (**A**) journals and (**B**) co-citation journals.

Subsequently, we screened 30 journals with at least two publications to visualize the journal network (Fig. **5A**). According to the thickness of the links between different nodes, a significant citation relationship existed among the journals at different times. Stem Cell Research & Therapy had an active citation relationship with Cells, Frontiers in Endocrinology, Stem Cells International, Reproductive Sciences, *etc*.

The co-citation frequency is another essential parameter for measuring the quality and impact of a journal. We conducted a co-citation journal analysis and mapped the co-citation visualization network using VOSviewer (Fig. **5B**), with a minimum of 50 citations. The results showed that the total number of co-citation journals was up to 1605. According to the statistics of the top 15 journals with the highest number of citations, these journals were mainly distributed in Q1 and Q2, and all had more than 130 citations. As shown in Table **4**, among the top 15 co-citation journals, five were cited more than 200 times. The Journal of Stem Cell Research & Therapy (Co-citation=1039) was the most cited, followed by Human Reproduction (Co-citation=373), Fertility and Sterility (Co-citation=325), Plos One (Co-citation=251), and Stem Cells (Co-citation=205). As for the journal IF ranking, the IF of Nature was the highest (IF=64.80), followed by Human Reproduction Update (IF=13.3) and Stem Cell Research & Therapy (IF=7.5). As shown in Fig. (**5B**), the Journal of Stem Cell Research & Therapy had positive co-citation relationships with Reproductive Sciences, Stem Cells International, International Journal of Molecular Sciences, Stem Cell Reviews and Reports, *etc*. Human reproduction had positive co-citation relationships with fertility and sterility, human reproduction updates, reproduction, biology of reproduction, *etc*. In addition, positive co-citation relationships existed between Plos One, Stem Cells, BioMed Research International, Stem Cells and Development, Journal of Translational Medicine, *etc*.

The dual-map overlay of journal analysis in CiteSpace was used to analyze the association of subject categories between citing and cited journals, standing for the citing trajectories for interdisciplinary collaboration and the dynamics of previous cross-discipline study activities [26]. The spline wave depicted the citation line, with citing journals on the left and cited journals on the right. As shown in Fig. (**6**), two primary citation lines tinted in orange described the main citation path in this field, which represented a large proportion of publications published in Molecular/Biology/Immunology journals, most likely cited publications from the Molecular/Biology/Genetics and Health/Nursing/Medicine journals.

### 3.5 Author and Co-citation Author Analysis

**Fig. (7):**
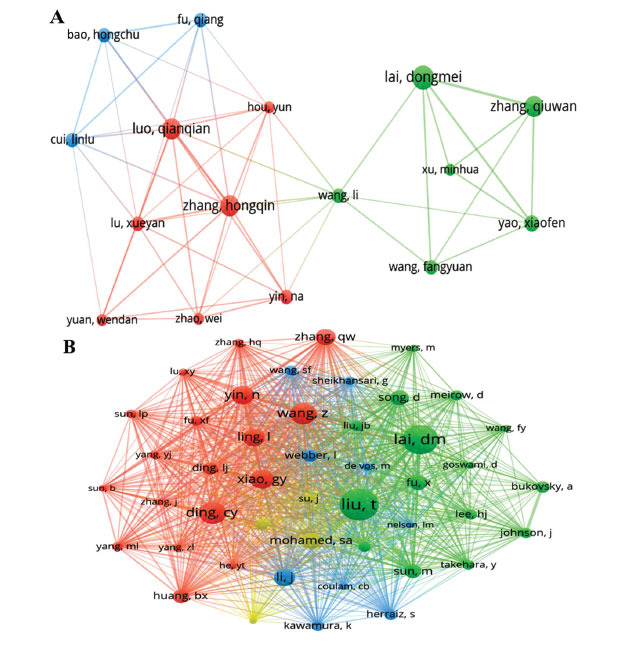
Visualization of (**A**) authors and (**B**) co-citation authors. (VOSviewer).

From the perspective of authors, a total of 1090 authors contributed to the research on stem cell therapy for POI. The top 10 authors published more than five papers each, with Lai D leading with 10 articles, followed by Li H and Huang B with 9 articles, indicating their fruitful contributions to the development of this field. Authors with three or more publications were incorporated in an author collaboration network analysis using VOSviewer. The result showed that a total of 68 authors were obtained, and 16 authors remained after excluding single authors (Fig. **7A**). Notably, the size of nodes in the collaboration network correlated with the number of publications, while the connecting lines represented collaboration among authors. They were mainly distributed in three clusters:blue cluster, red cluster, and green cluster, showing close collaboration among multiple authors. Fig. (**7A**). Lai D, Zhang Q, Luo Q, and Zhang H published the most related publications, so the nodes in the figure were the largest. The blue cluster featured close cooperation among Bao H, Cui L, and Fu Q. In terms of the red cluster, Luo Q had active cooperation with Zhang H, Lu X, Yin N, *etc*. Besides, we observed that Lai D had close collaborative relationships with Zhang Q, Yao X, Wang L, *etc*. The red cluster was tightly associated with the blue cluster, connected to the green cluster solely through Wang L.

In addition, the relationship among co-cited authors was analyzed. Among the 5560 co-cited authors, 11 authors were cited more than 60 times. Liu T was co-cited 129 times, ranking first, followed by Lai D (118 co-citations) and Ding C (89 co-citations), displaying their significant influence in the area of stem cell therapy for POI (Table **5**). Further, a total of 46 authors with minimum co-citations equal to 25 times were filtered to map the co-citation network diagram (Fig. **7B**). The collaboration analysis of core authors, labeled with different colors, revealed four main clusters using VOSviewer. The size of the nodes reflected the number of publications, while links indicated the density of authors' collaborations. Fig. (**7B**) illustrates active collaborations among co-cited authors, including Ding C, Lai D, Yin N, and Xiao G. The maps showed Liu T and Lai D centrally located in the green cluster, Li J in the blue cluster, Mohamed SA in the yellow cluster, as well as Yin N, Wang Z, Ling L, Xiao G, and Ding C in the red cluster. The close collaboration and communication among different clusters in the domain were acknowledged.

### 3.6 Co-citation Reference Analysis

**Fig. (8):**
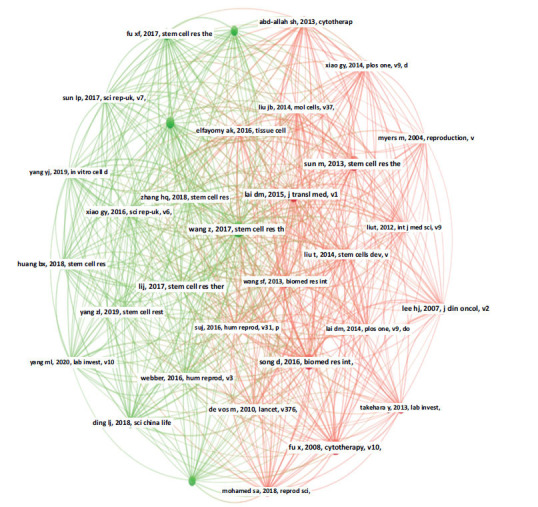
Visualization of co-citation references.

To effectively establish a foundation for research on stem cell therapy for POI, we performed a co-citation analysis of the cited documents in this field. To date, there have been a total of 6966 co-citation references. The top 10 co-citation references were all co-cited at least 41 times, with most being published in Stem Cell Research & Therapy. The top ten co-citation references with the largest number of citations were Wang Z (2017; 60 citations), Song D (2016; 59 citations), Fu X (2008; 58 citations), Sun M (2013; 55 citations), Lai DM (2015; 52 citations), Liu T (2014,45 citations), Ding LJ (2018,41 citations), Elfayomy AK (2016,41 citations), Lee HJ (2007,41 citations), and Ling L (2019,41 citations) [41-50]. Except for “Song D, 2016, Biomed Res Int”, which was published without an IF, the others were published in Q1 and Q2 journals, with IF ranging from 2.60 to 45.30. Subsequently, we constructed a co-citation network map for references with a minimum of 30 co-citations. In total, 33 references reached the threshold and were divided into two clusters (Fig. **8**). The size of the nodes corresponded to the frequency of references, and the line thickness represented the co-citation frequency. According to Fig. (**8**), “Wang Z, 2017, Stem Cell Res Ther” displayed active co-citation relationships with “Lai DM, 2015, J Transl Med”, “Li J, 2017, Stem Cell Res Ther” and “Song D, 2016, Biomed Res Int”, *etc*.

### 3.7 References with Citation Bursts

**Fig. (9):**
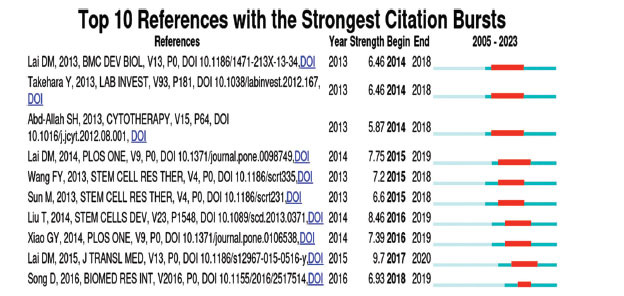
Top 10 references with the strongest citation bursts.

Notably, the references frequently cited by scholars in a certain field in a period were called the reference with citation bursts. In our study, the top 10 references in the field of stem cell therapy for POI with the strongest citation bursts were identified by CiteSpace (Fig. **9**). The cyan line represents the timeline, while the red bar within it indicates the duration of the citation outburst. Citation bursts for references appeared as early as 2014 and extended as late as 2020. As listed in Table **6**, we also summarized the citation burst strength, author, and title of each article in the order of the strength. The burst strength of the top 10 references ranged from 5.87 to 9.70, with durations lasting from 2 to 5 years. The reference with the most substantial citation burst (strength=9.70) was “Human endometrial mesenchymal stem cells restore ovarian function through improving the renewal of germline stem cells in a mouse model of premature ovarian failure,” authored by Lai D *et al*. and published in Journal of Translational Medicine, with citation bursts from 2017 to 2020. The second strongest citation burst (strength=8.46) was for the paper “Transplantation of human menstrual blood stem cells to treat premature ovarian failure in a mouse model,” published in Stem Cells and Development and authored by Liu T *et al*., with citation bursts from 2016 to 2019. Moreover, the third strongest citation burst (strength=7.75) was “Skin-derived mesenchymal stem cells help restore function to ovaries in a premature ovarian failure mouse model,” published *in* PLoS One and written by Lai D *et al*., with citation bursts from 2015 to 2019. Three of the top 10 references were completed by Lai D, including two in the top three, representing his significant influence in this field.

### 3.8 Keywords Co-occurrence Analysis

**Fig. (10):**
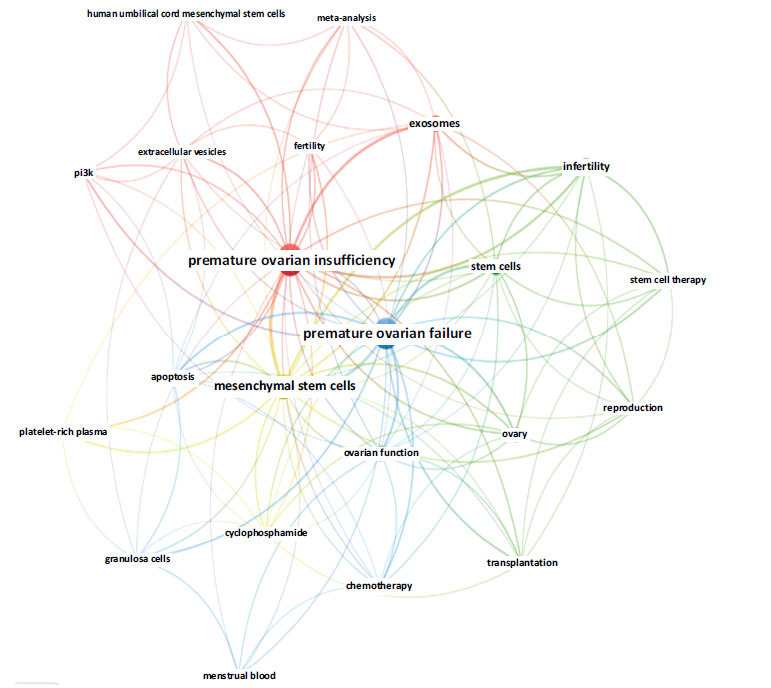
Visualization of co-occurrence keywords.

A total of 438 keywords were extracted from the retrieved publications. To effectively identify the research hotspots in the field of stem cell therapy for POI, VOSviewer was employed to conduct a co-occurrence analysis of keywords. Table **7** displays the ranking of the top 20 high-frequency co-occurrence keywords with a Total Link Strength (TLS) exceeding 10. Among these keywords, the five with the highest frequency were “premature ovarian failure” (70 times), “premature ovarian insufficiency” (69 times), “mesenchymal stem cells” (43 times), infertility (21 times), and “stem cells” (19 times), reflecting the main research direction of this field. Stem cell therapy for POI extends beyond stem cells themselves to include microvesicles or exosomes secreted by stem cells. Fertility in patients with POI has gradually attracted scholars’ attention. After merging keywords with similar meanings, we filtered those with a minimum occurrence threshold of five and performed cluster analysis using VOSviewer (Fig. **10**). The lines connecting the nodes illustrated the relationships among the keywords. Based on this network, similar keywords were categorized into four distinct clusters, each represented by a different color. The red cluster included exosomes, extracellular vesicles, human umbilical cord mesenchymal stem cells, P13K, premature ovarian insufficiency, fertility, and meta-analysis. Infertility, stem cells, stem cell therapy, transplantation, ovaries, and reproduction were grouped into green clusters. The keywords in the blue cluster consisted of apoptosis, chemotherapy, granulosa cells, menstrual blood, ovarian function, and premature ovarian failure. The remaining keywords in the yellow cluster included cyclophosphamide, mesenchymal stem cells, and platelet-rich plasma. Meanwhile, the core keywords “mesenchymal stem cells,” “premature ovarian failure,” and “premature ovarian insufficiency” were centrally located in the network.

### 3.9 Hotspots and Frontiers

**Fig. (11):**
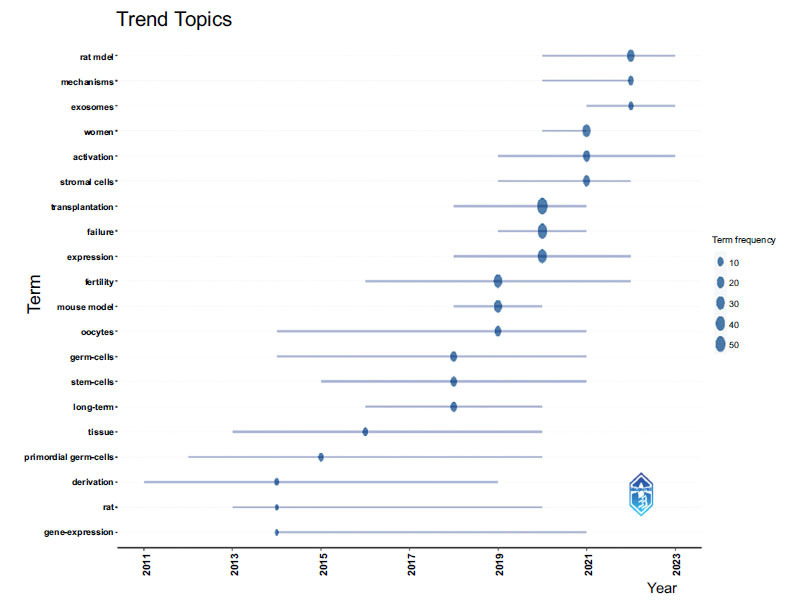
Hotspots and frontiers.

In this context, we used bibliometrix to analyze a trend topic analysis of the keywords, reflecting the changes in research hot topics over different periods (Fig. **11**). From 2011 to 2020, research predominantly focused on ovarian changes following stem cell transplantation, with the keywords including germ cells, oocytes, gene expression, tissue, and fertility. However, varying research directions existed during different periods. Since 2020, frequently cited terms have shifted to exosomes and mechanisms, indicating that the research on stem cell therapy for POI has expanded beyond the stem cells themselves to include exosomes produced by stem cells and their potential therapeutic mechanisms. They likely represent the current research hotspots and may become the frontier of research frontier in the near future.

## 4 DISCUSSION

In contrast to traditional systematic reviews, bibliometric analysis systematically evaluates and visualizes scholarly literature within a specialized field.

In this study, we conducted a bibliometric analysis of the literature published in the field of stem cell therapy for POI using the WOSCC database from January 1, 2000, to August 28, 2023, employing three bibliometric visualization software tools. A total of 203 publications were retrieved, published in 93 journals by 1090 authors affiliated with 321 institutes across 27 countries. This studysummarizes the trends in annual publications, citation bursts for references, research hotspots, and frontiers. It includes an analysis of countries, institutions, journals, and authors, as well as a co-citation analysis of journals, authors, references, and keywords on stem cell therapy for POI based on bibliometric analysis. The study aims to provide a reference point and predict the forefront hotspots in this field.

According to the trend of annual publications in stem cell therapy for POI, it can be divided into three stages: the initial stage (2000-2012), the development stage (2013-2017), and the vigorous stage (2018-2023). The first article in the field of stem cell therapy for POI was published in 2005, indicating that foundational research on the relationship between stem cell therapy and POI had not yet commenced. In the development stage, the field experienced slow growth, with an average of 6.2 publications per year. In the vigorous stage, the number of publications increased, reaching an annual average of 33.4 publications, significantly surpassing the combined output of the previous two phases. Furthermore, publications from the past five years accounted for 75.86% of all identified records, indicating that this research is in a period of rapid expansion, attracting considerable scholars’ attention to exploring stem cell therapy for POI. Our results suggested that research on stem cell therapy for POI demonstrated a rapidly developing trend, especially in 2021, reflecting the increasing interest and emphasis on this therapy within the academic community. Based on the current growth trend, the number of related articles on stem cell therapy for POI is expected to continue to rise, suggesting that this field holds significant potential for attracting new researchers.

A total of 27 countries around the world conducted research on stem cell therapy for POI, with half of the most productive countries located in Asia. In terms of national contributions, China accounted for 67.00% of the total publications (n=136), ranking first, and had the highest number of citations (n=3995), far ahead of the United States (618 citations), Iran (359 citations), and other countries. This finding indicated that China played a leading role in stem cell therapy-related research for POI. We further analyzed the average citations among countries with four or more publications and observed that Japan, China, and the United States ranked as the top three countries with average citations of 40.00, 29.38, and 24.72, respectively. These findings suggested that the number of publications and citations alone did not fully represent academic influence; therefore, it was necessary to evaluate the academic impact of a country’s scientific research achievements with average citations. However, there might be bias due to the relatively small number of publications. Regarding inter-country cooperation, we noticed different degrees of cooperation among countries, with China beingthe most productive country, establishing the strongest links with several countries, especially the United States and Australia. Therefore, these countries might continue to play a leading role in future research on stem cell therapy for POI. Nevertheless, compared with other fields of research, overall international collaboration in this area remains limited. Thus, it was essential to enhance efforts to promote academic productivity and strengthen collaborations with other countries in the field of stem cell therapy for POI.

When considering research institutions, 11 of the top 15 were located in China, while the remaining 4 were situated in the United States, Iran, and the Czech Republic, respectively. Regarding academic achievements, Shanghai Jiao Tong University in China ranked first for the number of publications (n=18), citations (n=837), and average citations (n=46.5). It was followed by Nanjing Medical University (n=16, 573, and 35.8, respectively) and Binzhou Medical University (n=9, 272, and 30.22, respectively), indicating that these institutions were leading the charge in this field. There were good cooperative relationships between some institutions. As the most productive institution, Shanghai Jiao Tong University has actively collaborated with several well-known universities, including Tongji University, Soochow University, Shanghai University of Traditional Chinese Medicine, Second Military Medical University, *etc*. However, the breadth and intensity of cooperation between institutions in China and those in other countries did not meet expectations, potentially hindering the long-term development of this research field. Therefore, it is suggested that extensive cross-institutional cooperation be pursued among research institutions across different countries to facilitate further study and promote the development of stem cell therapy for POI.

In terms of journals, Stem Cell Research & Therapy, which published 43 studies related to stem cell therapy for POI, was the most popular journal for researchers in this field, followed by Reproductive Sciences (n=9) and Stem Cell Reviews and Reports (n=7). Publication statistics substantially provided researchers with an efficient means to track academic frontiers and advancements in stem cell therapy for POI. Among the journals, the one with the highest IF and citations was Stem Cell Research & Therapy (IF=7.5, Q1, 1624 citations), the core journal in this field, while Scientific Reports had the highest average citation (60.75 times). Regarding the co-cited journals, Stem Cell Research & Therapy held the top spot with a total of 1,039 citations. Both the productivity ranking and the co-cited periodical ranking primarily comprised high-impact Q1 and Q2 journals. However, current research on stem cell therapy for POI is mainly published in journals related to biology, experimental science, and tissue engineering rather than in clinically oriented publications, indicating that the field remained largely in the basic experimental stage. Moreover, high-quality international journals are more likely to be favored by researchers, both in terms of paper submissions and literature references. The analysis of journal productivity and co-citation provides detailed and accurate guidance for researchers in the field of stem cell therapy for POI, aiding in the study and submission of relevant articles in the future.

As the three most prolific authors in the field of stem cell therapy for POI, Lai D ranked first with ten articles, followed by Li H and Huang B, who are tied for second with nine articles each. Of the seven articles, Lai D focused on the treatment of POI by human Amniotic Epithelial Cells (hAECs), while the remaining three revealed the roles of human endometrial stromal cells, skin-derived mesenchymal stem cells, and human amniotic fluid mesenchymal stem cells (hAFMSCs) in improving ovarian function, respectively [45, 51-53]. Lai D has revealed the therapeutic efficacy and main pathways of hAECs and their derivatives (conditioned medium, exosomes) in the treatment of POI through paracrine pathway secretion of a variety of factors that improve the ovarian microenvironment, thereby regulating apoptosis, inflammation, and angiogenesis [54-56]. The sodium alginate-bioglass composite hydrogel has the potential to further stimulate the paracrine effect of hAECs, thereby maximizing their therapeutic effect [57].

Li H and Huang B, affiliated with the same institution, have collaborated closely, co-authoring 8 of the 9 articles. In five co-authored articles, they described the potential of fetal liver mesenchymal stem cells, human Placental Mesenchymal Stem Cells (hPMSCs), human Amniotic Mesenchymal Stem Cells (hAMSCs), hAECs, and hAFMSCs for the treatment of POI [58-61]. In addition, they found that, due to the unique biological characteristics of hAMSCs, these cells outperformed hAECs in the treatment of POI and that the application of vitamin C could promote ovarian function in hAMSCs [60, 62]. The remaining three articles, co-authored by the two authors, focused on exosomes. In the treatment of POI, miR-320a in the hAMSC-derived Exosomes (hAMSC-Exos) regulated SIRT4, thereby reducing apoptosis and reactive oxygen species levels [63]. Human Adipose Stem Cell-derived Exosomes (hADSC-Exos) upregulated the SMAD signaling pathway, contributing to the restoration of ovarian function [64]. MiR-17-5p of human Umbilical Cord Mesenchymal Stem Cell-derived Exosomes (hUCMSC-Exos) inhibited SIRT7 expression, promoted cell proliferation, and reduced oxidative stress damage [65]. In two solo-authored articles, Li H explored the potential of hUCMSCs therapy for POI to restore ovarian endocrine function and follicle development while also reducing ovarian cell apoptosis [42]. Huang B found that exosomes of Menstrual blood-derived Stromal Cells(MenSC-Exos] exhibited the same therapeutic effect on POI as those of MenSCs and that the injection of MenSC-Exos was safer than that of MenSCs [66]. The three authors mentioned above have actively conducted a series of basic studies on stem cell therapy for POI. Building upon these studies, they have conducted stem cell-derived exosome therapy for POI, thereby establishing a solid foundation for further research and facilitating its clinical translation.

Co-cited references can be considered as basic research in a field [29]. In this study, the ten publications with the highest number of co-citations were listed as foundational research in the field of stem cell therapy for POI. Nine of the ten most highly cited publications were preclinical studies, with one classified as a clinical study. The preclinical studies represent the earliest investigations in the field of stem cell therapy for POI, exploring the therapeutic effects of various sources of stem cells, including BMSCs, MenSCs, endometrial stromal cells, hUCMSCs, and hAMSCs. As the most co-cited literature, Wang *et al*. verified that MenSCs repaired the ovaries damaged by chemotherapy and improved ovarian function, possibly by increasing the secretion of FGF2 [41]. These studies demonstrated that various sources of stem cells could improve ovarian function in animal models of POI without significant adverse events, thereby affirming the efficacy and safety of stem cell therapy in this context. Numerous studies indicate that stem cells predominantly exert their effects *via* the paracrine pathway. For instance, Sun *et al*. found that both intravenous and ovarian injections enhanced ovarian function, while Ding *et al*. combined umbilical cord MSCs with collagen scaffolds to synergistically promote the repair of ovarian function in patients with POI, facilitating successful clinical pregnancies [44, 47]. These findings indicate that stem cells from diverse sources possess significant potential for the treatment of POI, offering new insights and methodologies that lay a foundation for further clinical research and the development of therapeutic strategies.

Reviewing the top ten most influential studies in the field of stem cell therapy for POI reveals that all studies are preclinical and represent some of the earliest investigations, with several being the first to apply different types of stem cells to animal models of POI. The results demonstrated that four pieces of literature in study 1, 3, 5, and 8 were co-authored by Lai D, while study 2 was co-authored by Liu T, and study 6 was co-authored by Li H. The specific contents of the aforementioned research have been described in the previous section. In study 4, Xiao *et al*. indicated that hAFMSCs could prevent follicular atresia and preserve the fertility of POF mice; however, this therapeutic effect was not achieved through the differentiation of hAFMSCs into Granulosa Cells (GCs) or germ cells [67]. Studies 7 and 9 demonstrated that hADSCs restored ovarian function through altering alterations in gene expression, cytokine secretion, reduction of granulosa cell apoptosis, increased follicle numbers, and promotion of angiogenesis [44, 68]. Study 10 revealed that BMSCs inhibited the expression of apoptosis-related genes and secreted VEGF to promote the repair of ovarian function [69]. Nearly all studies utilized the number of follicles and hormone levels as indicators of ovarian function improvement to validate the effectiveness of various stem cell sources in treating POI. Four of the studies further validated the enhancement of fertility in POI animal models following stem cell therapy [45, 52, 67, 68]. The study by Takehara *et al*. and Xiao *et al*. observed the development of offspring, finding that all developed normally without malformations or adverse events, thereby providing evidence for the safety of stem cell therapy in the context of POI [67, 68]. In addition to exploring safety and efficacy, the researchers conducted a preliminary investigation into the mechanisms of stem cell therapy, discovering that transplanted stem cells can promote angiogenesis, inhibit apoptosis, reduce inflammation, and differentiate into granulosa cells to facilitate follicular development. This suggests that stem cells operate synergistically through multiple pathways to enhance the recovery of ovarian function. The success of the preclinical studies demonstrates the feasibility of stem cell therapy for POI and establishes a solid foundation for further research and clinical trials.

We analyzed keywords to capture the distribution and evolution of hotspots and trends in the field of stem cell therapy for POI. The results revealed that stem cell-derived exosome therapy and its related mechanisms represent the current hotspots and frontiers in this field. Stem cells, with their potential for self-renewal and multidirectional differentiation, play vital roles in repairing damaged tissues and maintaining cellular homeostasis, demonstrating significant potential for a variety of intractable diseases [70]. Although stem cell therapy is currently regarded as one of the most promising and popular treatment modalities, concerns regarding its immunogenicity and tumorigenic risks associated with long-term use have been raised by numerous scholars. In recent years, with the gradual deepening of research, scholars have found that the biological effects of stem cells were mainly through paracrine pathways, especially *via* exosomes. Exosomes are extracellular vesicles measuring 40 to 160 nm (average 100 nm) that encompass various cellular components, including DNAs, RNAs, lipids, metabolites, cytoplasm, and cell surface proteins, facilitating information transfer [71]. Exosomes derived from stem cells exhibit biological functions analogous to those of stem cells and have emerged as a novel therapeutic strategy for a variety of refractory diseases. For instance, in autoimmune encephalomyelitis, exosome therapy has been shown to reduce neuroinflammation, regulate T cells, and promote sustained clinical recovery [72]. Exosomes enriched with a variety of growth factors, proteins, and miRNAs have been found to reverse insulin resistance, alleviate β cell apoptosis, and restore insulin secretion in the treatment of diabetes and its complications [73]. In the treatment of intrauterine adhesions, exosomes have been shown to promote vascular regeneration, repair endometrial injuries, and enhance fertility, with their efficacy being comparable to that of stem cells [74].

At present, several basic and preclinical studies on stem cell-derived exosome therapy for POI have been conducted both domestically and internationally. Common sources of exosomes include hUCMSCs, BMSCs, embryonic stem cells, hAFMSCs, hADSCs, and MenSCs. Zhang *et al*. confirmed that repeated application of exosomes derived from MenSCs could reverse chemotherapy-induced POI, with no significant difference in therapeutic effect between exosomes and MenSCs [66]. Similarly, BMSC-derived Exosomes (BMSCs-Exos) restored ovarian function and fertility in POI mice without adversely affecting their offspring. Although the efficacy and duration of action of exosomes are slightly lower than those of stem cells, this can be compensated by increasing the doses and administering multiple injections [75]. The efficacy of exosomes from different stem cell sources for the treatment of POI has been confirmed by several scholars. With the continuous deepening of research, scholars have begun to explore the molecular mechanisms by which exosomes treat for POI. Research indicates that the improvement of ovarian function by hADSC-Exos occurs through the upregulation of the SMAD pathway [64]. Table **8** lists the miRNAs involved in stem cell-derived exosome therapy for POI, along with their associated pathways. As the most widely used cell types in regenerative medicine, the therapeutic effects of hUCMSCs for POI have been affirmed by many researchers.As research advances, researchers are progressively exploring the corresponding therapeutic mechanism. Basic experiments have demonstrated that the therapeutic effects of hUCMSCs are closely related to their paracrine functions. HUCMSC-Exos contains a variety of miRNAs, including miR-17-5p, miR-29a, miR-126-3p, and miR-22-3p. miR-145-5p can restore ovarian function by alleviating oxidative damage and apoptosis in GCs while promoting GC proliferation and angiogenesis [65, 76-78]. In the treatment of POI, BMSCs recovered the estrus cycle in rats, improved sex hormone levels, decreased apoptosis in GCs, prevented ovarian follicular atresia, and increased the number of follicles [79]. This therapeutic effectis largely due to its paracrine effect. BMSC-Exos up-regulated miR-144-5p, miR-644-5p, and circLRRC8A, thereby inhibiting apoptosis in GCs and enhancing ovarian function [79-81]. HAFMSC-derived Exosomes (hAFMSC-Exos) are enriched with a variety of miRNAs, such as miR-10a, miR-146a, and miR-369-3p, which inhibit apoptosis-related genes, thereby inhibiting apoptosis in GCs and protecting follicles [82, 83]. Therefore, the treatment of POI with exosomes occurs through multiple molecular pathways. To fully elucidate these mechanisms, extensive future research is necessary.

Exosomes secreted by stem cells, which exhibit therapeutic effects similar to those of their parent cells, are expected to replace stem cell therapy as a new therapeutic strategy for cell-free therapy in regenerative medicine. In addition to this, exosomes possess unique advantages, including their lack of a nucleus, which mitigates the risk of tumorigenesis. Various proteins and RNAs enriched in exosomes can target regulatory molecules [84]. Compared with stem cells, exosomes are easier to preserve, and long-term storage doesn’t affect their efficacy. The dosage, titer, and safety of exosomes can be evaluated similarly to conventional drugs, thereby facilitating their clinical application [85]. Exosomes, due to their smaller diameters, don’t form emboli that could obstruct blood vessels, penetrate the blood-brain barrier more efficiently, and can be modified to achieve higher titers [86]. Despite the numerous advantages of exosomes as cell-free therapies, several challenges remain, including efficient separation and purification methods, sourcing, optimal concentration, elucidation of specific molecular mechanisms, and ensuring the safety of exosomes in future applications.

### 4.1 Safety and Efficacy of Stem Cell Therapy for POI

Most current research on stem cell therapy for early-onset ovarian insufficiency focuses on preclinical studies. Studies have utilized chemotherapeutic agents to construct animal models of POI, employing various sources of stem cells, including embryonic stem cells, mesenchymal stem cells, and induced pluripotent stem cells for treatment. No adverse events were reported in numerous preclinical studies, thereby demonstrating the safety of stem cell therapy for POI. The efficacy of stem cell therapy for POI has been thoroughly validated in these studies. Stem cells from various sources induced the resumption of the estrous cycle, increased follicle counts at all stages and reproductive hormone levels, and improved fertility to varying degrees in animal models of POI. The exploration of mechanisms by researchers further provides substantial scientific evidence for the effectiveness of stem cell therapy for POI. In treating POI, stem cells often act through multiple pathways, such as regulating proliferation and apoptosis genes to promote Granulosa Cell (GC) proliferation and inhibit GC apoptosis, secreting various angiogenesis-related factors (*e.g*., VEGF, HGF) to synergistically promote angiogenesis, inhibiting the secretion of pro-inflammatory factors to mitigate inflammation, interacting with immune cells to regulate the inflammatory microenvironment, and inhibiting the production of reactive oxygen species while modulating oxidative stress [13, 87, 88].

### 4.2 Clinical Transformation of Stem Cell Therapy for POI

The success of stem cell therapy in animal models of POI provides compelling evidence to support the development of subsequent clinical trials. Herraiz *et al*. conducted a pilot study on the intra-ovarian transplantation of autologous bone marrow MSCs for the treatment of POI. The study utilized elevated sinus follicle counts and anti-Müllerian hormone levels as indicators of improvement in ovarian function, finding that 81.3% of the patients experienced an enhancement in ovarian function, with five successfully conceiving [89]. Yan *et al*. performed hUCMSCs transplantation on 61 patients with POI, and no serious side effects or complications related to the treatment were found during the follow-up. Most patients experienced follicle growth in different stages within the ovaries following treatment, and two patients regained normal menstruation. The study also represented that the therapeutic effect of hUCMSCs was positively correlated with the number of antrum follicles before transplantation [17]. In addition to simple stem cell transplantation, Ding *et al*. used hUCMSCs in combination with collagen scaffoldings for the treatment of POI patients, which resulted in an increase in the number of follicles, promoted follicle development, and improved hormone levels and ovarian function [47]. The safety, efficacy, and feasibility of hADSCs for treating POI patients have also been proven, and studies have shown that two transplants of 5×10^6^ hADSCs achieved the best therapeutic effect [19]. Zafardoust *et al*. carried out autologous MenSCs ovarian transplantation in POI patients and found that the symptoms, such as vaginal dryness, excessive sweating, and persistent hot flashes, were significantly improved. Additionally, some patients resumed menstruation, FSH levels were reduced, and ovarian function was repaired [18].

### 4.3 Prospects for Future Research

Over the past two decades, numerous studies have assessed the effectiveness of stem cell therapy from various aspects, including follicular development, hormone levels, and fertility, as well as the safety of stem cell therapy regarding the occurrence of adverse events and offspring development. Additionally, these studies have elucidated the mechanisms of stem cell therapy from multiple perspectives, such as angiogenesis, inflammation regulation, apoptosis, and differentiation. These breakthroughs have rendered clinical translation feasible and expanded the prospects for stem cell therapy in the field of POI; however, significant challenges remain. Currently, there is a paucity of studies comparing the advantages and disadvantages of different sources of stem cells for POI regarding the preparation process, yield, and therapeutic efficacy; future research must address this gap. Combination therapy represents a vital avenue for future research, integrating non-toxic, biocompatible materials with stem cells for the treatment of POI to enhance the efficacy of stem cell therapy and identify optimal therapeutic options. Although preclinical studies have demonstrated that stem cells enhance ovarian function through various pathways, extensive research is required to explore the specific signaling pathways and mechanisms underlying stem cell therapy for POI. Currently, there is no established standard for the application of stem cells in the treatment of POI; thus, further studies are essential to investigate the optimal dosage, timing, route, and frequency of stem cell therapy to achieve improved therapeutic outcomes. Safety and efficacy continue to be focal points of future research, necessitating large-scale clinical trials to validate the long-term safety and effectiveness of stem cell therapy.

### 4.4 Practical Significance and Limitations

Through bibliometrics, this study analyzed the field of stem cell therapy for POI, objectively summarizing the most productive countries, institutions, and authors, as well as listing the most popular journals and literature. A detailed analysis of the research status of stem cell therapy for POI and the forefront of the field's hotspots was presented in chart form for scholars. By reading this article, scholars can efficiently identify the countries, institutions, authors, journals, and literature related to stem cell therapy for POI, enabling them to pursue their research interests more effectively.

This study also presents several limitations. First, our study only included the WOSCC database and did not extract or analyze commonly used medical databases such as PubMed and Embase. Second, by establishing the screening criteria, studies published in non-English languages were excluded, potentially introducing bias by ignoring relevant research. Third, the predetermined research extraction timeframe limited the inclusion of new articles published after this date. Fourth, a certain period is required for articles to accumulate citations, resulting in some high-quality articles lacking sufficient citations due to their relatively recent publication.

## CONCLUSION

As the most promising treatment for POI, stem cell therapy has high research value and application prospects. This study used three bibliometric software tools, including VOSviewer, CiteSpace, and Bibliometricx, to analyze the current status of stem cell therapy for POI from the perspectives of countries, institutions, journals, authors, references, and keywords and to predict the hotspots and frontiers in this field. Further analysis of stem cell therapy for POI demonstrated that stem cell-derived exosome therapy and its corresponding mechanisms may represent future research hotspots. With the increasing attention of researchers toward basic experiments in stem cell therapy for POI, its clinical transformation has garnered significant interest. Adult-derived hUCMSCs, BMSCs, hADSCs, and MenSCs have been used in phase 1 clinical trials. Although the safety and efficacy of stem cell therapy for POI have been preliminarily verified in a Phase 1 clinical trial, further trials are necessary to validate its future clinical rollout on a global scale.

## Figures and Tables

**Fig. (6):**
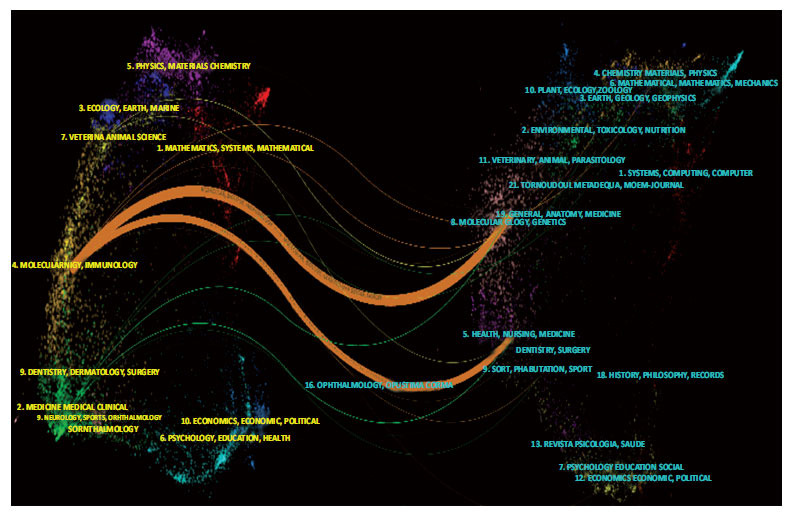
Dual-map overlay of journals.

**Table 1:** Top 10 countries in stem cell therapy for POI.

**Rank**	**Country**	**Distribution**	**Count**	**Citations**	**Average Citations**
1	China	Asia	136	3995	29.38
2	The United States	North America	25	618	24.72
3	Iran	Asia	22	359	16.32
4	Egypt	Africa	6	148	24.67
5	South Korea	Asia	6	143	23.83
6	Spain	Europe	6	109	18.17
7	Turkey	Asia	6	83	13.83
8	Australia	Oceania	4	78	19.50
9	Italy	Europe	4	35	8.75
10	Japan	Asia	4	160	40.00

**Table 2:** Top 15 research universities and institutions in stem cell therapy for POI.

**Rank**	**Universities and Institutions**	**Country**	**Count**	**Citation**	**Average Citations**
1	Shanghai Jiao Tong University	China	18	837	46.50
2	Nanjing Medical University	China	16	573	35.81
3	Binzhou Medical University	China	9	272	30.22
4	Chongqing Medical University	China	9	193	21.44
5	Chinese Academy of Sciences	China	8	342	42.75
6	Tabriz University of Medical Sciences	Iran	8	156	19.50
7	Zhejiang University	China	8	237	29.63
8	Shanghai University of Traditional Chinese Medicine	China	7	289	41.29
9	Huazhong University of Science and Technology	China	6	87	14.50
10	Shandong University	China	6	131	21.83
11	Tongji University	China	6	246	41.00
12	University of Illinois	The United States	6	116	19.33
13	Charles University	The Czech Republic	5	130	26.00
14	Soochow University	China	5	191	38.20
15	The University of Chicago	The United States	5	82	16.40

**Table 3:** Top 12 journals for research of stem cell therapy for POI.

**Rank**	**Journal**	**Count**	**Q**	**IF**	**Citation**	**Average Citation**
1	Stem Cell Research & Therapy	43	Q1	7.50	1624	37.77
2	Reproductive Sciences	9	Q2	2.90	133	14.78
3	Stem Cell Reviews and Reports	7	Q2	4.80	125	17.86
4	Frontiers in Endocrinology	6	Q1	5.20	73	12.17
5	Journal of Ovarian Research	5	Q1	4.00	60	12.00
6	Stem Cells International	5	Q2	4.30	126	25.20
7	Biomed Research International	4	Q3	3.25	197	49.25
8	Cell Transplantation	4	Q2	3.30	47	11.75
9	Current Stem Cell Research & Therapy	4	Q4	2.70	37	9.25
10	Frontiers in Cell and Developmental Biology	4	Q1	5.50	25	6.25
11	Reproductive Biology andEndocrinology	4	Q1	4.40	114	28.50
12	Scientific Reports	4	Q2	4.60	243	60.75

**Table 4:** Top 15 co-citation journals for research of stem cell therapy for POI.

**Rank**	**Co-citation Journal**	**Citation**	**Q**	**IF**
1	Stem Cell Research & Therapy	1039	Q1	7.50
2	Human Reproduction	373	Q1	6.10
3	Fertility and Sterility	325	Q1	6.70
4	Plos One	251	Q2	3.70
5	Stem Cells	205	Q1	5.20
6	Reproductive Sciences	183	Q2	2.90
7	Cytotherapy	172	Q2	4.50
8	Biology of Reproduction	169	Q2	3.60
9	Human Reproduction Update	166	Q1	13.30
10	BioMed Research International	165	Q3	3.25
11	Reproduction	165	Q2	3.80
12	Stem Cells and Development	165	Q2	4.00
13	Scientific Reports	164	Q2	4.60
14	Stem Cells International	157	Q2	4.30
15	Nature	132	Q1	64.80

**Table 5:** Top 10 authors and co-citation authors on the research of stem cell therapy for POI.

**Rank**	**Authors**	**Count**	**Co-citation Authors**	**Co-citations**
1	Lai D	10	Liu T	129
2	Li H	9	Lai D	118
3	Huang B	9	Ding C	89
4	Ding C	8	Wang Z	85
5	Zou Q	8	Xiao G	77
6	Zhang H	8	Yin N	76
7	Luo Q	8	Ling L	72
8	Zhang Q	8	Li J	69
9	Lu J	6	Zhang Q	66
10	Al-Hendy A	6	Mohamed SA	65

**Table 6:** Top 10 references with the strongest citation bursts.

**Rank**	**Strength**	**Author**	**Title**
1	9.70	Lai D	Human endometrial mesenchymal stem cells restore ovarian function by improving the renewal of germline stem cells in a mouse model of premature ovarian failure
2	8.46	Liu T	Transplantation of human menstrual blood stem cells to treat premature ovarian failure in mouse model
3	7.75	Lai D	Skin-derived mesenchymal stem cells help restore function to ovaries in a premature ovarian failure mouse model
4	7.39	Xiao G	Amniotic fluid stem cells prevent follicle atresia and rescue fertility of mice with premature ovarian failure induced by chemotherapy
5	7.20	Wang F	Human amniotic epithelial cells can differentiate into granulosa cells and restore folliculogenesis in a mouse model of chemotherapy-induced premature ovarian failure
6	6.93	Song D	Human umbilical cord mesenchymal stem cells therapy in cyclophosphamide-induced premature ovarian failure rat model
7	6.60	Sun M	Adipose-derived stem cells improved mouse ovary function after chemotherapy-induced ovary failure
8	6.46	Lai D	Human amniotic fluid stem cells have the potential to recover ovarian function in mice with chemotherapy-induced sterility
9	6.46	Takehara Y	The restorative effects of adipose-derived mesenchymal stem cells on damaged ovarian function
10	5.87	Abd-Allah SH	Mechanistic action of mesenchymal stem cell injection in the treatment of chemically induced ovarian failure in rabbits

**Table 7:** Top 20 co-occurrence keywords on the research of stem cell therapy for POI.

**Rank**	**Co-occurrence** **Keywords**	**Counts**	**Total Link Strength**
1	Premature ovarian failure	70	99
2	Premature ovarian insufficiency	69	91
3	Mesenchymal stem cells	43	92
4	Infertility	21	40
5	Stem cells	19	33
6	Exosomes	17	36
7	Ovarian function	16	32
8	Ovary	14	26
9	Apoptosis	13	24
10	Chemotherapy	11	17
11	Stem cell therapy	10	18
12	Transplantation	9	17
13	Cyclophosphamide	8	18
14	Granulosa cells	8	16
15	PI3K	7	12
16	Extracellular vesicles	6	16
17	Fertility	6	14
18	Reproduction	6	16
19	Meta-analysis	5	13
20	Platelet-rich plasma	5	12

**Table 8:** Mechanisms and pathways of miRNAs in exosomes for treatment of POI.

**Source**	**Cargo**	**Pathway**	**Function**	**References**
HUCMSCs	miR-17-5P↑	SIRT7/ (PARP1, γH2AX, XRCC6)	Reactive oxygen species accumulation↓	[56]
HUCMSCs	miR-29a↑	HBP1/Wnt/β-Catenin	Proliferation↑, Apoptosis↓	[70]
HUCMSCs	miR-126-3p↑	PIK3R2/PI3K/AKT/mTOR	Proliferation↑, Apoptosis↓, Angiogenesis↑	[71]
HUCMSCs	miR-22-3p↑	KLF6/ATF4-ATF3-CHOP	Apoptosis↓	[78]
HUCMSCs	miR-145-5p↑	XBP1	Oxidative injury↓, Apoptosis↓	[72]
BMSCs	miR-144-5p↑	PTEN/PI3K/AKT	Apoptosis↓	[73]
BMSCs	miR-644-5p↑	p53	Apoptosis↓	[74]
BMSCs	circLRRC8A↑	miR-125a-3p/NFE2L1	Oxidative damage↓	[75]
HAFMSCs	miR-146a↑miR-10a↑	-	Apoptosis↓	[76]
HAFMSCs	miR-369-3p↑	YAF2/PDCD5/p53	Apoptosis↓	[77]

## LIST OF ABBREVIATIONS

BMSCs: Bone Marrow Mesenchymal Stem Cells
BMSCs-Exos: Bone Marrow Mesenchymal Stem Cell-derived Exosomes
ESHRE: European Society for Human Reproduction and Embryology
GCs: Granulosa Cells
hADSC-Exos: Human Adipose Stem Cell-derived Exosomes
hADSCs: Human Adipose Stem Cells
hAECs: Human Amniotic Epithelial Cells
hAFMSC-Exos: Human Amniotic Fluid Mesenchymal Stem Cell-derived Exosomes
hAFMSCs: Human Amniotic Fluid Mesenchymal Stem Cells
hAMSC-Exos: Human Amniotic Mesenchymal Stem Cell-derived Exosomes
hAMSCs: Human Amniotic Mesenchymal Stem Cells
hPMSCs: Human Placental Mesenchymal Stem Cells
hUCMSC-Exos: Human Umbilical Cord Mesenchymal Stem Cell-derived Exosomes
hUCMSCs: Human Umbilical Cord Mesenchymal Stem Cells
MenSC-Exos: Menstrual Blood-derived Stromal Cell-derived Exosomes
MenSCs: Menstrual Blood-derived Stromal Cells
POF: Premature Ovarian Failure
WOS: Web of Science
WOSCC: Web of Science Core Collection
